# Waste to Wealth: Electrochemical Innovations in Hydrogen Production From Industrial Wastewater

**DOI:** 10.1002/gch2.202500043

**Published:** 2025-04-25

**Authors:** Tesfaye Alamirew Dessie, Lemlem Seyoum Seifu, Woldesenbet Bafe Dilebo

**Affiliations:** ^1^ Faculty of Chemical and Food Engineering Bahir Dar Institute of Technology Bahir Dar University Bahir Dar 79 Ethiopia; ^2^ College of Natural and computational Science Department of Chemistry Jinka University Jinka 5555 Ethiopia

**Keywords:** alcohol oxidation reactions, amine oxidation reaction, hydrazine oxidation reactions, iodine oxidation reaction, urea oxidation reactions, wastewater

## Abstract

The increasing demand for energy and the environmental challenges posed by fossil fuel consumption prompts the exploration of clean and sustainable energy solutions. This review article focuses on the innovative approach of generating energy through the electrolysis of wastewater, which not only facilitates clean energy production but also aids in wastewater treatment. Significant advancements in electrooxidation processes for the sustainable production of hydrogen and other valuable chemicals are highlighted. This article specifically analyzes the techno‐economic aspects of electrooxidation for small molecules, including alcohol, amine, hydrazine, iodine, and urea, within the framework of wastewater treatment. Cost estimations for hydrogen and value‐added products derived from the oxidation reactions are presented, with production costs calculated at $6.37, $6.06, $2.68, $5.69, and $10.69 per kilogram of H_2_, respectively. However, the costs associated with alcohol oxidation reactions and urea oxidation reactions are deemed unfeasible. An analysis of profitability reveals that the oxidation processes for iodine, hydrazine, and amine wastewater generate revenue profits of 28%, 16%, and 6%, respectively.

## Graphical abstract

There is increasing worry regarding the environmental impact of waste from hospital sewage, agricultural runoff, and industrial wastewater. Those wastes can contaminate water ecosystems, harming both human health and aquatic life. By employing an electrochemical method, we can treat these toxic substances in wastewater while simultaneously producing hydrogen gas and other valuable byproducts, which can generate income.

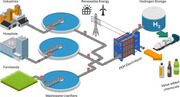

## 1 Introduction

The rising demand for energy and the worsening environmental pollution resulting from fossil fuel consumption have driven researchers to explore clean and sustainable energy sources derived from wastewater electrolysis. Hydrogen (H_2_) is a clean energy carrier that offers high energy density and produces no carbon emissions, presenting a potential solution to the energy crisis our society is currently experiencing.^[^ ^1^ ^]^ A major problem is that currently, ≈95% of hydrogen production relies on fossil fuel reforming, which releases carbon dioxide and is very damaging to the environment.^[^ ^2^ ^]^ This reality highlights the urgent need to develop effective and environmentally sustainable hydrogen production methods. Consequently, significant resources have been dedicated to exploring eco‐friendly techniques for hydrogen production, with water electrolysis being acknowledged as one of the most promising methods.^[^ ^3^ ^]^ This technique generates pure hydrogen without carbon emissions, representing a major advancement in sustainable industrial production.^[^ ^4^ ^]^ The widespread adoption of hydrogen production through water electrolysis faces significant obstacles, primarily due to its high energy requirements and related costs. Recent data indicates that the production costs of hydrogen derived from fossil fuels, such as natural gas reforming, range from $1.30 to $1.50 per kilogram. In contrast, the process of generating hydrogen through electrochemical water splitting is significantly more costly.^[^ ^5^ ^]^ Even with progress, there are still ongoing technical problems, especially the slow reaction rates and high energy demands in water electrolysis. The large amount of energy needed, caused by the high thermodynamic barrier and slow kinetics of the four‐electron‐transfer oxygen evolution reaction (OER), remains a major challenge.^[^ ^6^ ^]^ The voltage needed for traditional water splitting usually goes above 1.5 V, but the theoretical potential for this process is only 1.23 V.^[^ ^7^ ^]^ It is also important to note that the water‐splitting byproduct at the anode, oxygen gas, holds considerably less economic value.^[^ ^8^ ^]^ This presents an alternative method for reducing energy consumption by replacing the OER with anodic oxidation reactions involving small molecules that exhibit lower thermodynamic barriers.^[^ ^9^ ^]^ The electro‐oxidation of various small organic compounds, including urea, alcohols, amines, hydrazine, and iodine, has been suggested to lower the overall cell voltage.^[^ ^10^ ^]^ Concerns regarding the environmental impacts of Amine, Alcohol, Hydrazine, Iodine, and Urea wastes have increased, with research indicating that many of these substances can infiltrate aquatic ecosystems, leading to harmful effects on both humans and aquatic life due to their continuous discharge from domestic sewage, agricultural runoff, and industrial waste water.^[^ ^11^ ^]^ Conventional wastewater treatment facilities frequently do not succeed in fully removing these contaminants.^[^ ^12^ ^]^ An electrochemical method has been utilized as a successful means to remove different types of pollutants, relying on a complex mechanism influenced by the characteristics of electrodes and electrolytes. Electrochemical techniques for wastewater treatment offer several benefits compared to traditional methods, such as the absence of the need for additional chemicals, reduced sludge production, compact equipment ideal for installation and automation, and, most significantly, the generation of hydrogen gas and other valuable byproducts.^[^ ^13^ ^]^

This review significant advancements in electrooxidation processes for the sustainable production of hydrogen and other valuable chemicals are highlighted and the latest advancements in electrocatalysts designed for the oxidation of alcohols, amines, hydrazine, iodine, and urea in the context of hydrogen production are reviewed. Subsequently, a techno‐economic analysis is performed on the reviewed electro catalysts and small molecules. The review concludes with a brief discussion of potential future strategies.

## 2 Electro‐Oxidation of Wastewater to Produce H_2_

**Figure 1:**
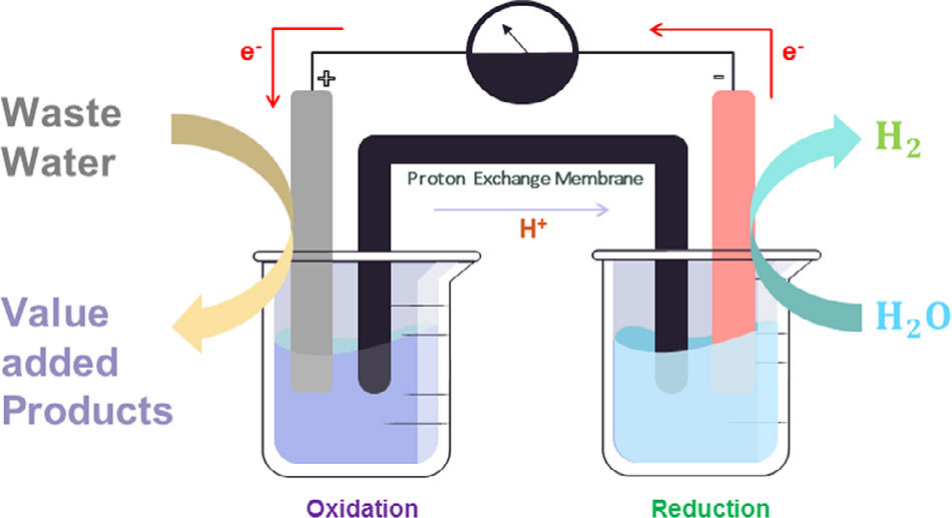
The schematic diagram for electrochemical oxidation of wastewater to value‐added products.

Water electrolysis technology aims to use less energy while producing more hydrogen. However, commercial water electrolyzers struggle with high energy demand. These challenges show the need for new methods to make hydrogen production more efficient and sustainable.^[^ ^14^ ^]^ The electrooxidation process of wastewater emerges as a novel technique for hydrogen production, while simultaneously serving as a wastewater treatment solution. The schematic diagram illustrating overall water splitting through wastewater electrolysis is shown in **Figure** **1**. This approach has the potential to yield higher‐quality water suitable for reuse and to sustainably transform wastes into valuable products (**Table** **1**).^[^ ^15^ ^]^

**Table 1:** Selected wastewater for anodic oxidation reactions.

Molecules	Bond energy [kJ mol^−1^]	Standard Potentials [V vs RHE]	Overall redox reaction	Value added products
Water (H_2_O)	O─H: 498.7	1.23	H_2_O → H_2 _+ O_2_	H_2_
Methanol (CH_3_OH)	C─H _methyle_:401.9 O─H: 440.2	0.04	CH_3_OH + H_2_O → HCOOH + 2H_2_	HCOOH, H_2_
Ethanol (CH_3_CH_2_OH)	C─H _methylene_:396.6	0.05	CH_3_CH_2_OH + H_2_O → CH_3_COOH + 2H_2_	CH_3_COOH, 2H_2_
Amine (RNH_2_)	N─H: 301.2	−0.77	R_3‐N + H_2_O → R_2‐NH^+ ^+ H_2_	H_2_, *n*‐butyronitriles, Octenenitriles, Benzonitrile, Imine.
Hydrazine (NH_2_─NH_2_)	N─H: 338.1	−0.33	N_2_H_4_ → N_2_ + 2H_2_	H_2_
Iodide	–	0.53	6I^− ^+ 4 H_2_O → I_2_ + 4 H_2_ + $I_{3}^{-}+IO_{4}^{-}$	I_2,_ H_2_
Urea (CO(NH_2_)_2_)	N─H: 464.4	0.37	CO(NH_2_) + H_2_O	N_2_, CO_2_, 3H_2_

**Figure 2:**
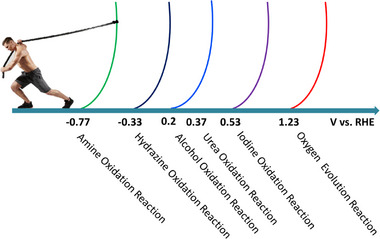
Thermodynamic equilibrium potentials of various oxidation processes in wastewater electrolysis.

Given their importance and the research related to them for hydrogen generation through electrolysis, the focus will be directed toward five key types of wastewater: Alcohol, Amine, Hydrazine, Iodide, and Urea. The anodic OER is a slow process that requires four electrons and one proton, necessitating a high overpotential (1.23 V vs RHE) and the use of a precious electrocatalyst to enable water dissociation.^[^ ^15^ ^]^ The sluggish nature of the anodic OER primarily contributes to increased energy consumption, and the oxygen generated at the anode is often not needed in practical applications.^[^ ^9^ ^]^ A recent energy‐efficient approach to hydrogen production has been introduced, replacing the traditional anodic OER with alternative oxidation agents sourced from wastewater, such as the oxidation of alcohols, amines, hydrazine, iodine, and urea. Hydrazine, amine, urea, and iodide each have thermodynamic oxidation potentials of −0.33, −0.77, 0.37, and 0.53 V versus RHE, respectively (**Figure** **2**).^[^ ^16^ ^]^

### 2.1 Alcohol Oxidation Reaction (AOR)

Alcohols are organic substances that have one or more hydroxyl groups linked to a carbon atom. Wastewater from distilleries frequently has high levels of organic substances, including alcohol. The flammability and potential toxicity of these compounds make them potentially dangerous. They have the potential to pollute water sources and disturb ecosystems if improperly handled.^[^ ^17^ ^]^ Due to the wastewater's hazardous nature, effective treatment procedures that can break down these organic pollutants are required. Electrochemical anodic oxidation is a highly effective approach for treating distillery wastewater. This technique offers a practical solution for addressing the challenges posed by distillery wastewater that contains toxic alcohol. In addition to breaking down harmful compounds, this method generates hydrogen gas and other valuable byproducts, providing both environmental and economic benefits.^[^ ^18^ ^]^

The methanol oxidation reaction proceeds as follows:^[^ ^19^ ^]^ (Equations (1)–(5)

(1)

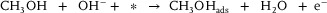



(2)

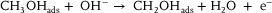



(3)

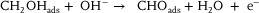



(4)

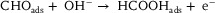



(5)

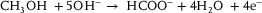




Similarly, ethanol oxidation yields acetic acid and hydrogen gas. The ethanol oxidation reaction proceeds as follows:^[^ ^20^ ^]^ (Equations (6)–(11)

(6)

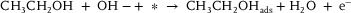



(7)

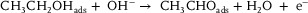



(8)

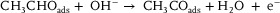



(9)

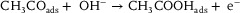



(10)

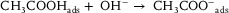

(11) $$\;CH_{3}CH_{2}\mathrm{OH}\;+5OH^{-}\to \;CH_{3}\mathrm{CO}O^{-}+4H_{2}O$$

**Figure 3:**
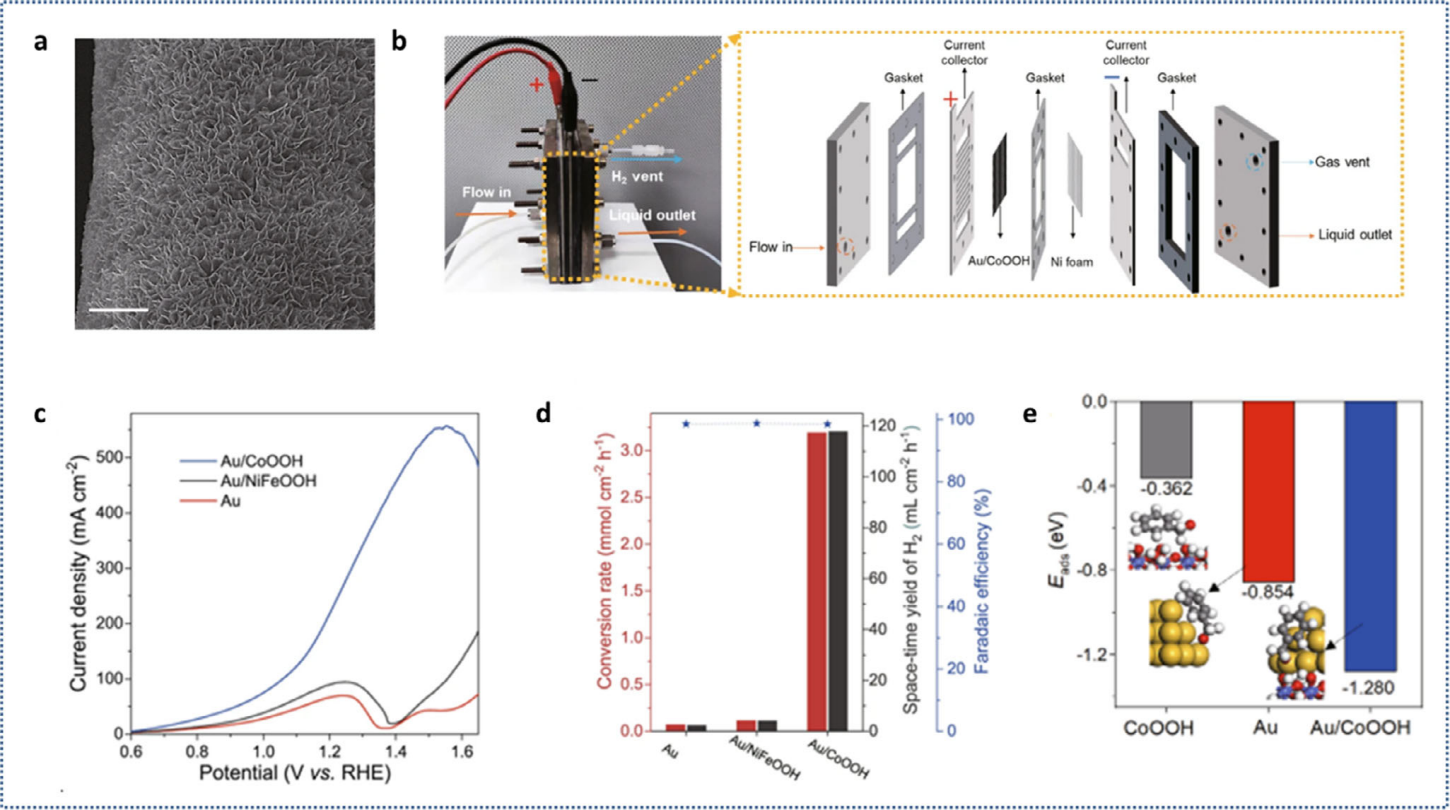
a) Scanning electron microscopy (SEM) image of Au/CoOOH. b) Electrolyzer operating without a membrane for free flow. c) Linear sweep voltammetry (LSV) curves for Au/CoOOH, Au/NiFeOOH, and Ni foam‐supported Au at a scan rate of 10 mV s^−1^ in a 1 m KOH solution containing 0.1 m benzyl alcohol. d) Conversion rates of benzyl alcohol, hydrogen production yield, and Faradaic efficiency measured in a 1 m KOH solution with 0.1 m benzyl alcohol at 1.3 V relative to the reversible hydrogen electrode (RHE), utilizing various catalysts alongside a platinum cathode. e) Energies of alkoxide adsorption for benzyl alcohol on CoOOH, Au, and Au/CoOOH, respectively. Reproduce with permission,^[^ ^21^ ^]^ Copyright (2022) The Author(s).

Recent studies have demonstrated the effectiveness of catalysts such as Au/CoOOH for alcohol oxidation, achieving high current densities and hydrogen production rates. Li et al. developed a cooperative catalyst made of gold nanoparticles (NPs) on cobalt oxyhydroxide nanosheets (Au/CoOOH) to achieve high activity for H_2_ production at high current density (**Figure** **3**a). In a practical two‐electrode membrane‐free flow electrolyzer, the current could reach 4.8 A at 2 V, demonstrating the catalyst's capability for industrial applications (Figure 3b). At 1.5 V versus RHE, the current density could rise to 540 mA cm^−2^, marking the highest known value for such a low potential (Figure 3c). When tested at a constant potential of 1.3 V versus RHE, the rates for benzyl alcohol oxidation and H_2_ generation reached 3.19 mmol cm^−2^ h^−1^ and 117.9 mL cm^−2^ h^−1^, respectively, which are 26 and 28 times higher than those of pure gold. The Au/CoOOH catalyst showed a current density of 340 mA cm^−2^ at 1.3 V versus RHE in a solution of 1 m KOH and 0.1 m benzyl alcohol at room temperature (Figure 3d). Experiments, along with spin‐polarized density functional theory (DFT) analysis (Figure 3e), showed that benzyl alcohol (as alkoxide) accumulates at the Au/CoOOH interface and is oxidized by the electrophilic OH* generated on CoOOH, leading to better performance compared to pure gold.^[^ ^21^ ^]^

### 2.2 Amine Oxidation Reaction (AmOR)

Amines are nitrogen‐rich compounds that originate from ammonia, distinguished by their diverse structural types and important functions in both chemical and biological contexts.^[^ ^22^ ^]^ In industrial applications, amines are utilized in the manufacturing of dyes, medications, rubber, and synthetic resins, among various other uses.^[^ ^23^ ^]^ The primary origin of amine emissions is the carbon capture and storage sector. Within this sector, amines serve as solvents for the absorption of carbon dioxide (CO_2_) from flue gases emitted by industries. During this operation, a certain quantity of the amine solvent can be lost due to various factors such as evaporation, thermal breakdown, or reactions with other elements in the gas stream. This leads to the release of amines into wastewater discharges, which must be treated before being released.^[^ ^24^ ^]^

Amines can be oxidized and converted to nitriles. C─N bonds are activated and dehydrated to form C≡N bond or C═N bond, depending on the catalyst used. Amine oxidation products may include *n*‐butyronitriles, octenenitriles, benzonitrile, and imine.^[^ ^25^ ^]^

Amines are harder to oxidize than alcohols because different products can form based on the oxidants and conditions used. To turn an amine into an imine, a dehydrogenation reaction is needed. Converting primary amines to nitriles is even more difficult.^[^ ^26^ ^]^ Nitrile functional groups are important in many active compounds, leading to a growing interest in finding cheap, effective, and eco‐friendly ways to produce nitriles. Imine compounds are just as crucial as amines and are essential in agriculture, medicine, and chemical biology. Electrochemical oxidation is a promising method for treating wastewater that has amine groups.^[^ ^27^ ^]^

Primary amines oxidation to nitriles proceeds as follows (Equations (12)–(15)):^[^ ^28^ ^]^

(12) $$R-NH_{2}+*\to *R-NH_{2}$$

(13) $$*R-NH_{2}\to R-\mathrm{NH}*$$

(14) $$R-\mathrm{NH}*\to R-C\equiv N+H^{+}+e^{+}$$

(15) $$R-NH_{2}\to R-C\equiv N+H^{+}+e^{+}$$

The secondary amines oxidation to imines proceeds as follows (Equations (16)–(18)):^[^ ^29^ ^]^

(16) $$R_{1}R_{2}\mathrm{NH}+\;*\to R_{1}R_{2}\mathrm{NH}*$$

(17) $$R_{1}R_{2}\mathrm{NH}*\to R_{1}R_{2}C\;=NR_{3}+H^{+}+e^{-}$$

(18) $$R_{1}R_{2}\mathrm{NH}\to R_{1}R_{2}C\;=NR_{3}+H^{+}+e^{-}$$

Tertiary amines (R_1_R_2_R_3_N) are more challenging to oxidize due to the absence of hydrogen atoms on the nitrogen. However, they can undergo oxidation to form amine oxides or other nitrogen‐containing compounds.

Waldvogel and his research team chose mesitylaldoxime as their substrate, with methyltriethylammonium methylsulfate acting as the electrolyte in acetonitrile. They used graphite and glassy carbon as anodes, while glassy carbon, lead, stainless steel, nickel, platinum, and boron‐doped diamond served as cathodes. This research demonstrated a simple, halogen‐free method to create nitriles from oximes at room temperature, using affordable and readily available electrode materials like lead and graphite in an undivided cell, achieving an isolated yield of up to 81% for aromatic nitriles.^[^ ^30^ ^]^

**Figure 4:**
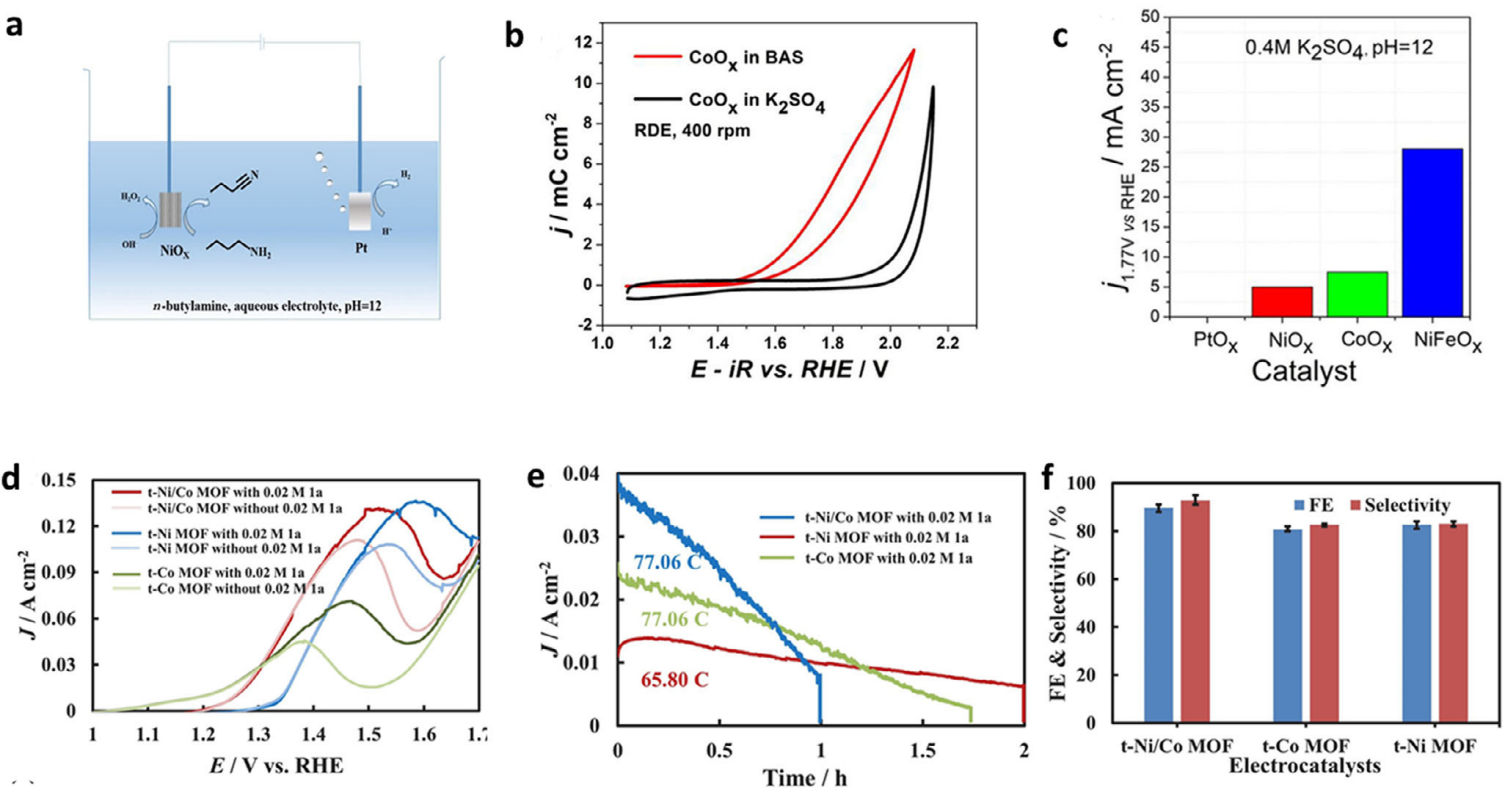
a) Diagrammatic representation of the oxidation process of *n‐*butylamine. b) Cyclic voltammetry (CV) data for CoOx thin films in a 0.4 m BAS and 0.4 m K_2_SO_4_ environment. c) Measurement of current densities at 1.77 V relative to the reversible hydrogen electrode (RHE) for various metal‐oxide electrodes in a 0.4 m K_2_SO_4_ electrolyte.^[^ ^27^ ^]^ Copyright 2017, Wiley−VCH d) LSV curves of the t‐Ni MOF, t‐Co MOF, and t‐Ni/Co MOF electrodes at a scan rate of 10 mV S^−1^ in 10 mL of 1 m KOH with or without 0.02 m 1a. e) Chronoamperometry experiments of the t‐Ni MOF, t‐Co MOF, and t‐Ni/Co MOF electrodes at 1.35 V. f) Faradaic efficiencies and selectivity of t‐Ni MOF, t‐Co MOF, and t‐Ni/Co MOF electrodes.^[^ ^32^ ^]^ Copyright 2022 Elsevier.

Xu et al. further note a marked decrease in the observed anodic overpotentials within the water electrolysis system when employing electrolytes that incorporate *n*‐butylamine in conjunction with catalysts that are widely used in contemporary industrial applications. Additionally, they successfully promoted the oxidation of butylamine to butyronitrile at a pH of 12, utilizing a 0.4 m BAS electrolyte (**Figure** **4**a,d,e).^[^ ^31^ ^]^

M. Xiang et al. developed an oxidant‐free method for the selective conversion of primary amines into nitriles using the bimetallic Ni/Co metal–organic framework derivative (t‐Ni/Co MOF) as the anodic electrocatalyst. Aromatic and aliphatic nitriles can be efficiently synthesized with high yields and faradaic efficiencies using the t‐Ni/Co MOF electrode in conjunction with the hydrogen evolution reaction. Benzylamine may be oxidized to benzonitrile at a remarkably low potential of 1.3 V versus reversible hydrogen electrode (RHE) using the t‐Ni/Co MOF electrode, which is lower than the potentials reported for Ni‐based monometallic electrocatalysts in benzylamine electrooxidation (Figure 4b,c,f).^[^ ^32^ ^]^

### 2.3 Hydrazine Oxidation Reaction (HzOR)

Hydrazine is a colorless, combustible liquid that has a smell like ammonia. It is extremely dangerous unless it is managed in a solution, like hydrazine hydrate. It has various applications, including as a foaming agent, a building block for pharmaceuticals and agrochemicals, and as a propellant for spacecraft. Additionally, it serves as an oxygen scavenger in power plants to minimize corrosion.^[^ ^33^ ^]^ Industries that produce, handle, or utilize hydrazine, or that use it as fuel, have the potential to release hydrazine into the environment and industrial wastewater. This toxic chemical in wastewater should be broken down through electrochemical anodic oxidation, while simultaneously generating H_2_ at the cathode.^[^ ^34^ ^]^ To achieve large‐scale production of H_2_, through HER and HzOR can be used instead of OER.^[^ ^35^ ^]^ Anodic oxidation of hydrazine in aqueous solution is a four‐electron pathway and N_2_ is the final product. Even if the final product N_2_ is less important, Hydrazine degradation in the wastewater is significant to protect the environment from this hazardous waste.^[^ ^36^ ^]^

The hydrazine oxidation reaction follows a four‐electron pathway, producing nitrogen gas as the final product (Equations (19)–(24)). The limiting reaction is Equation (19).^[^ ^34^ ^]^

(19)

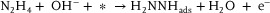



(20)

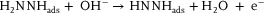



(21)

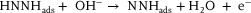



(22)

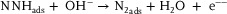

(23) $${N_{2}}_{\mathrm{ads}}\to N_{2}+\;*$$

(24) $$N_{2}H_{4}+4OH^{-}\to \;N2+4H_{2}O\;+4e^{-}$$

**Figure 5:**
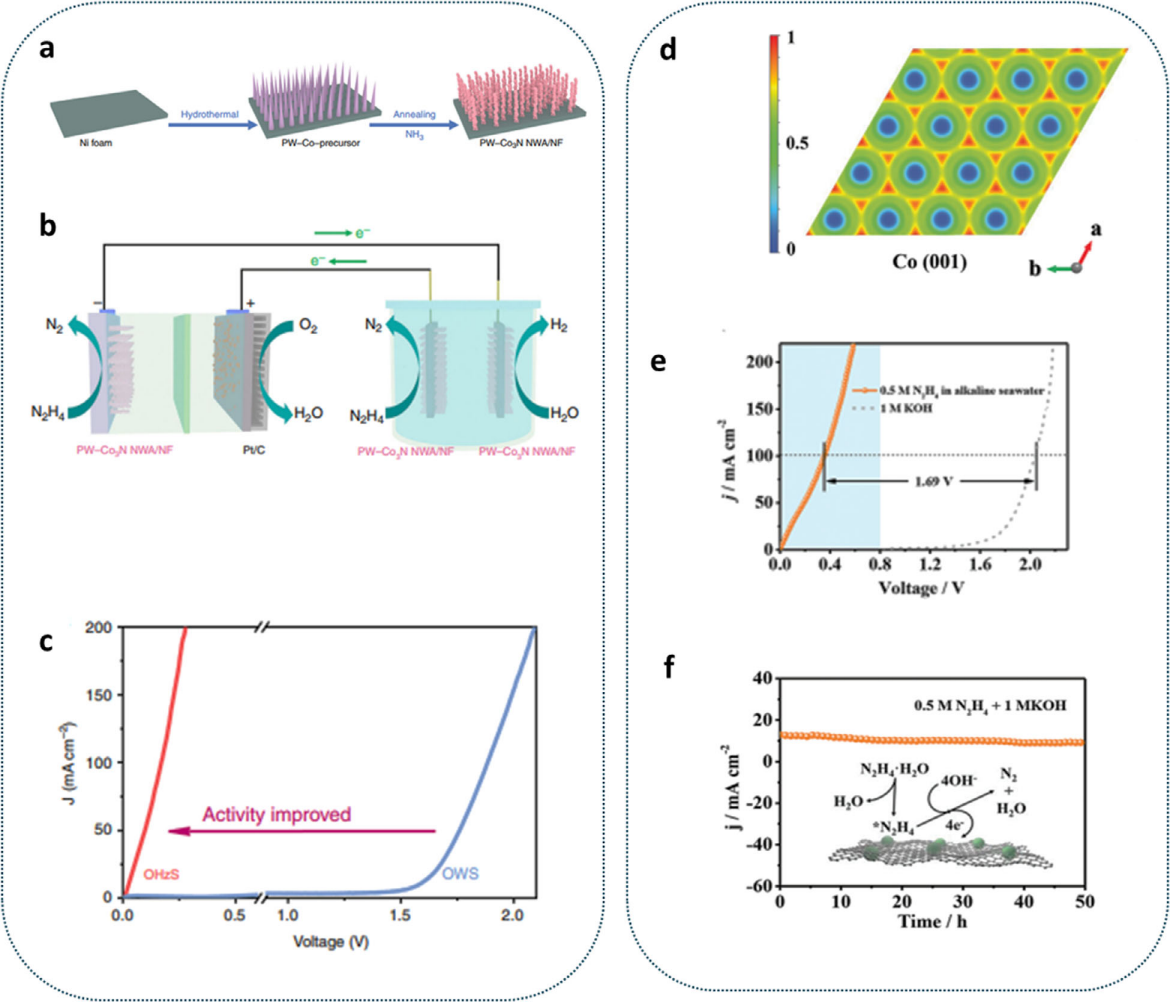
a) A schematic representation depicting the synthesis process of PW‐Co3N NWA/NF. b) A schematic representation illustrating a self‐sustaining hydrogen production system. c) Linear sweep voltammetry (LSV) curves for hydrogen oxidation reaction (HzOR) in a 1 m KOH/0.1 m hydrazine solution and oxygen evolution reaction (OER) in 1 m KOH, utilizing PW‐Co_3_N NWA/NF as both the anode and cathode. Reproduce with permission^[^ ^33^ ^]^ Copyright (2020), The Author(s) d) Maps of the electron localization function for Co (001) bifunctional CC@CoNC‐600 electrode. e) LSV curves for HzOR and OER on the CC@CoNC‐600 electrode. f) The stability assessment of the CC@CoNC‐600 electrode along with a schematic diagram illustrating the potential pathway for HzOR. cm^−2^.^[^ ^38^ ^]^ Copyright 2023, Wiley−VCH.

Liu et al. present a novel composite electrode composed of phosphorus and tungsten co‐doped Co_3_N nanowire arrays, which are synthesized in situ on nickel foam, designated as PW‐Co3N NWA/NF. This electrode demonstrates exceptional performance as a bifunctional electrocatalyst for both the hydrazine oxidation reaction (HzOR) and the hydrogen evolution reaction (HER) (**Figure** **5**a,b). Remarkably, it achieves current densities of 10, 200, and 600 mA cm^−2^ at working potentials of −55, 27, and 127 mV (vs RHE) for HzOR in a 1 m KOH/0.1 m N_2_H_4_ electrolyte, surpassing existing leading benchmarks (Figure 5c). DFT calculations indicate that the incorporation of phosphorus and tungsten significantly diminishes the free‐energy changes linked to the dehydrogenation of adsorbed hydrazine (*NH_2_NH_2_) and enhances the thermoneutrality of the free energy of adsorbed hydrogen (ΔGH*) in comparison to unmodified Co_3_N.^[^ ^37^ ^]^

Xin et al. presented the synthesis of cobalt/nitrogen‐codoped carbon (CoNC) nanosheet arrays on carbon cloth (CC) utilizing an efficient ZIF‐L‐templated method (Figure 5d). This innovative material functions as a high‐performance bifunctional electrocatalyst (CC@CoNC) for both the hydrogen evolution reaction (HER) and the hydrazine oxidation reaction (HzOR), exhibiting impressively low overpotentials of −69 mV for HER and −61 mV for HzOR to achieve a current density of 10 mA cm^−2^.(Figure 5e). Furthermore, a chlorine‐free and energy‐efficient system for hydrogen production has been developed using CC@CoNC, which has undergone pyrolysis at 600 °C (designated as CC@CoNC‐600) for both cathodic and anodic applications. The CC@CoNC‐600 electrode, due to its advantageous chlorine‐free characteristic, also exhibits remarkable stability in a hydrazine‐assisted electrolysis system utilizing seawater, maintaining reliable operation for over 40 h at a current density of ≈10 mA cm^−2^ (Figure 5f).^[^ ^38^ ^]^

### 2.4 Iodide Oxidation Reaction

Iodide, the anion of iodine (I^−^), has vital applications in medicine, nutrition, and industrial operations. Iodide can enter wastewater from a variety of sources, including industrial processes such as LCD manufacture, medical facilities, and natural sources. Understanding these sources can help in designing effective treatment strategies for iodide removal. Several iodide removal methods are used in wastewater treatment plants, including membrane separation, electrochemical oxidation, and adsorption.^[^ ^39^ ^]^ As illustrated in the reaction pathways below (Equations (25)–(32)), electrochemical oxidation presents a viable method for efficiently treating iodide in wastewater. Beyond purification, this process also yields valuable byproducts such as hydrogen and iodine.

The iodide oxidation reaction proceeds as follows (Equations (25)–(32)). The limiting step of this reaction is represented by Equation (26).^[^ ^40^ ^]^

(25) $$2I^{-}↔I_{2}+2e^{-}$$

(26) $$I_{2}+I^{-}↔I_{3}^{-}$$

(27) $$2I_{3}^{-}↔3I_{2}+2e^{-}$$

(28) $$I^{-}+3H_{2}O↔{\mathrm{IO}}_{3}^{-}+6H^{+}+6e^{-}$$

(29) $${\mathrm{IO}}_{3}^{-}+2OH^{-}↔{\mathrm{IO}}_{4}^{-}+H_{2}O+2e^{-}$$

(30) $$I^{-}+OH^{-}↔\mathrm{HOI}+2e^{-}$$

(31) $$\mathrm{HOI}+I^{-}+H^{+}↔I_{2}+H_{2}O$$

(32) $$6I^{-}+3OH^{-}+H_{2}O↔I_{2}+{\mathrm{IO}}_{4}^{-}+5H^{+}+14e^{-}$$

**Figure 6:**
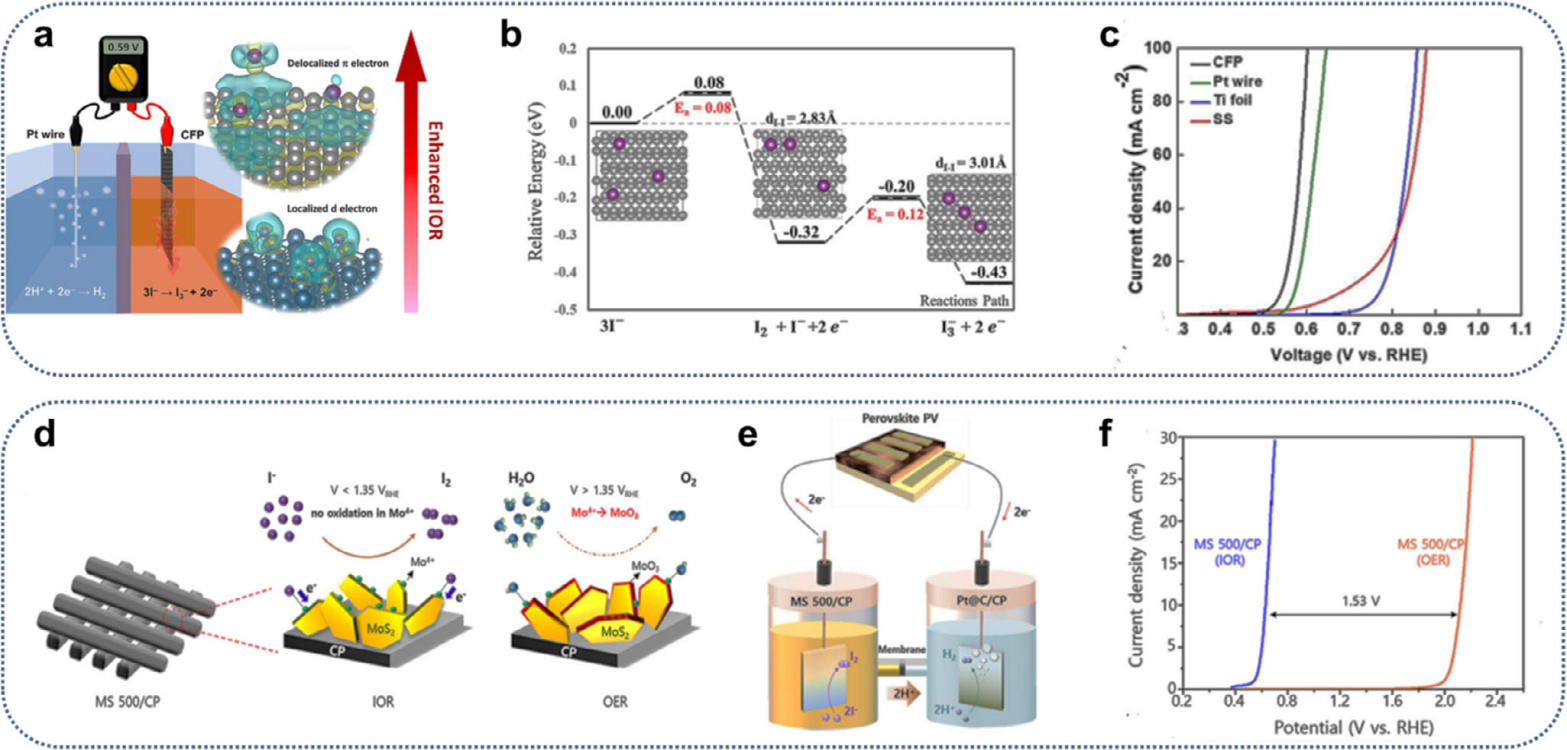
a) A schematic representation of the IOR on a graphite framework characterized by delocalized π electrons. b) The potential energy profiles across the surface of graphite. c) LSV curves for the CFP electrocatalyst during the IOR process.^[^ ^39c^ ^]^ Copyright 2025, Royal Society of Chemistry. d) A schematic illustration depicting the IOR and OER processes utilizing MoS_2_. e) A schematic representation of the IOR process employing a MoS_2_/C catalyst within a PV‐EC cell. f) LSV curves for the MoS_2_/C catalyst under both IOR and OER conditions in hydroiodic acid and sulfuric acid (0.5 m H_2_SO_4_).^[^ ^41^ ^]^ Copyright 2023, Wiley.

According to the research conducted by Peng, S.M., et al., graphite‐structured carbon fiber paper (CFP) was identified as the catalyst, exhibiting an onset potential of 0.54 volts (vs RHE) for the iodide oxidation reaction (IOR), as illustrated in **Figure** **6**a. Furthermore, CFP displayed remarkable stability during testing, maintaining performance for over 18 h at a current density of 10 mA cm^−2^. The authors first established that the graphite orbital system, along with the 3I^−^ configuration representing the most stable adsorption mode, facilitates effective charge transfer. Their computational analyses indicated that iodide ions can physically adsorb onto the graphite surface, transferring a charge of 0.83 eV to an undistorted structure. Subsequent DFT calculations revealed that iodine (I_2_) molecules can form through the dimerization of the adsorbed iodide ions, with a minimal energy barrier of 0.08 eV (Figure 6b). Additionally, the triiodide ion ($I_{3}^{-}$) can be generated by the interaction of the adsorbed I_2_ molecule with another nearby iodide ion, requiring an energy barrier of 0.12 eV. Notably, the entire reaction is exothermic, exhibiting a favorable reaction energy of ≈0.43 eV.^[^ ^39c^ ^]^ The thermodynamic oxidation potential of IOR is usually 0.53 V (vs RHE). They discovered that delocalized electrons in graphite‐based catalysts can speed up the rate of charge transfer, leading to better activity for the iodide oxidation reaction at a low potential of 0.54 V (vs RHE), which is very close to the standard potential (Figure 6c).

Park et al. propose a heterostructure catalyst that exhibits remarkable catalytic performance for the iodine oxidation reaction (IOR), wherein atomic layer deposition (ALD) MoS_2_ is uniformly applied to carbon paper (CP) serving as a 3D current collector (Figure 6d). The catalyst benefits from the intrinsic iodide oxidation capability of MoS_2_, as confirmed by computational analyses, along with the substantial surface area of the substrate. This heterostructure achieves an iodide oxidation current density of 10 mA  cm^−2^ at a reduced voltage of 0.63 VRHE, which is 1.53 V lower than the voltage required for the OER to achieve the same current density. In addition to its outstanding catalytic efficiency in iodide environments, ALD MoS_2_ demonstrates impressive durability, maintaining performance for 200 h without deactivation, attributed to the low oxidative potential applied to the catalyst. A two‐electrode electrolyzer was developed by combining a commercial Pt@C catalyst as the cathode for the hydrogen evolution reaction (HER) with the ALD MoS_2_‐based heterostructure as the anode for the IOR, resulting in a current density of 10 mA cm^−2^ at a minimal cell voltage of 0.66 V. Furthermore, the researchers successfully illustrate an unbiased solar‐to‐hydrogen conversion device by integrating this electrolyzer with a single perovskite photovoltaic cell, achieving a record current density of 21 mA cm^−2^ under sunlight (Figure 6e,f). Their findings present a clear and practical approach for sustainable hydrogen production through an innovative combination of a photovoltaic–electrochemical (PV–EC) system and the iodine oxidation reaction as an alternative oxidation pathway.^[^ ^41^ ^]^

### 2.5 Urea Oxidation Reaction (UOR)

Urea is a nitrogen‐based compound frequently utilized as a fertilizer in farming activities. When introduced to crops, urea can be transformed into ammonia and later into nitrate by soil microorganisms via a process referred to as nitrification. Nonetheless, not all the applied urea is taken up by plants; a considerable amount can seep into groundwater or flow into adjacent water bodies, which results in the creation of urea‐rich wastewater.^[^ ^42^ ^]^

The anodic oxidation of urea in wastewater represents a promising and effective strategy for managing nitrogenous waste. Anodic urea oxidation reaction (UOR) is carried out at 0.37 V versus RHE which is far below the theoretical thermodynamic conventional water dissociation potential of 1.23 V versus RHE.^[^ ^43^ ^]^

The urea oxidation reaction proceeds as follows (Equations (33)–(39)):^[^ ^44^ ^]^

(33)

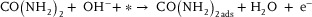



(34)

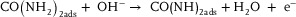



(35)

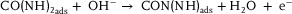



(36)

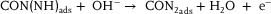



(37)

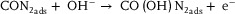



(38)

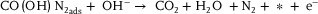



(39)

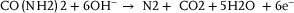




**Figure 7:**
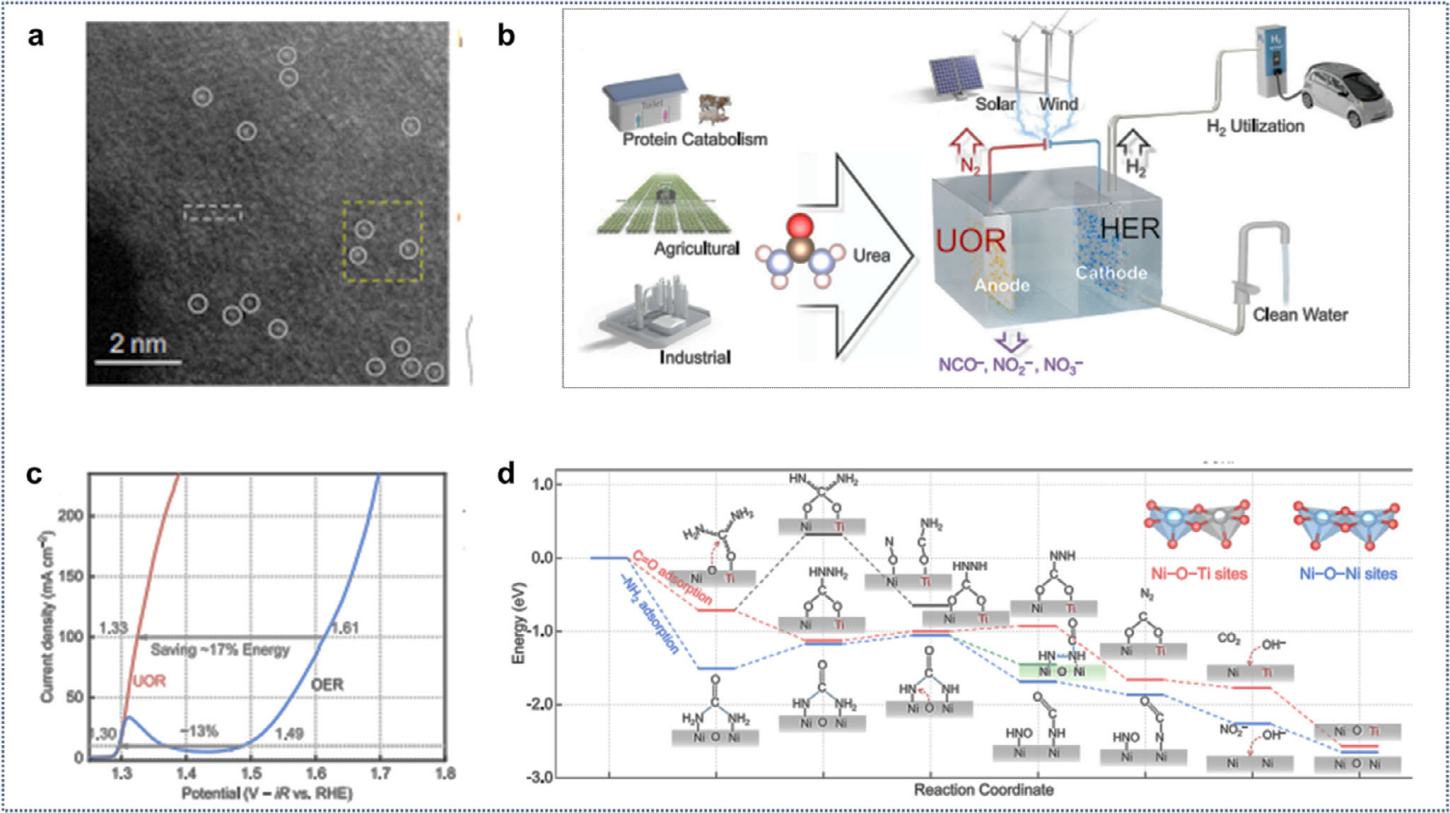
a) An atomic‐resolution HAADF‐STEM image depicting asymmetric Ni–O–Ti sites, with white circles indicating the presence of Ni atoms. b) A representation of the UOR process aimed at the production of clean water and energy. c) LSV curves demonstrating 85% iR correction for the asymmetric Ni–O–Ti sites in 1 m KOH combined with 0.33 m urea, as well as in 1 m KOH, for facilitating the UOR and OER. d) A diagram illustrating the UOR process alongside the Gibbs free energy variation for both asymmetric Ni–O–Ti and symmetric Ni–O–Ni sites. Reproduce with permission^[^ ^45^ ^]^ Copyright 2024, The Author(s).

Zhan et al. demonstrate that atomically isolated asymmetric Ni–O–Ti sites on a titanium foam anode achieve a nitrogen (N_2_) selectivity of 99% (**Figure** **7**a,b). This performance surpasses connected symmetric Ni–O–Ni counterparts found in previously reported nickel‐based electrocatalysts, which exhibit N_2_ selectivity below 55%. Additionally, these asymmetric sites yield a hydrogen (H_2_) evolution rate of 22 mL h^−1^ when coupled with a platinum counter cathode at a current density of 213 mA cm^−2^ and a voltage of 1.4 V versus the reversible hydrogen electrode (RHE) (Figure 7c). The presence of oxygenophilic titanium adjacent to nickel enhances the interaction with carbonyl groups rather than amino groups in urea, thus preventing premature cleavage of the resonant C═N bond before the intramolecular N–N coupling necessary for N_2_ production occurs (Figure 7d).^[^ ^45^ ^]^

## 3 Techno‐Economic Analysis

Wastewater electrolysis involves the utilization of electricity, water, and various small molecules, including alcohols, amines, hydrazine, iodide, and urea, as electrolytes. In evaluating wastewater electrolysis systems, it is crucial to account for several cost elements, such as expenses related to input chemicals (including electrolytes and buffers), capital investments in equipment (such as electrolyzers, hydrolyzers, and distillation apparatus), and electricity costs associated with the electrolysis and separation processes, and ongoing operational expenses.^[^ ^77^ ^]^ A thorough understanding and analysis of these diverse cost components are vital for enhancing wastewater electrolysis methods' efficiency and economic feasibility, thus facilitating the progress and implementation of green hydrogen production technologies in the quest for sustainable energy solutions.^[^ ^78^ ^]^

Value‐added water electrolysis differs from traditional methods that only produce hydrogen. It creates useful compounds by oxidizing different substrates. For example, amines can be transformed into amides, nitriles, amine oxides, oximes, imines, and azo compounds through electrochemical oxidation. These compounds are valuable in the pharmaceutical and agrochemical sectors.^[^ ^79^ ^]^ An additional significant criterion for assessing various oxidation reactions is the potential generation of value‐added chemicals. Controlling the reaction pathway to favor the most advantageous products is crucial, particularly for chemicals that undergo intricate competing oxidation reactions, as this can help minimize industrial purification and separation expenses.^[^ ^48^ ^]^

This review performs a detailed techno‐economic analysis to evaluate the economic viability of producing hydrogen on a large scale using wastewater electrolysis, based on a model created by the Sargent research group.^[^ ^80^ ^]^ This model provides a detailed estimate of production costs per ton of product, dividing these costs into three main types: raw materials, capital, and operating expenses. The initial capital cost is specifically for the electrolysis cell and does not include other equipment or infrastructure. The model assumes a factory lifespan of 30 years, with operating costs mainly driven by energy expenses, while also considering separation costs, factory operations, and other various expenses.

### 3.1 Material Cost

This study exclusively focuses on the costs associated with electrolytes, assuming that the feedstocks (namely amine, alcohol, hydrazine, iodine, and urea) are sourced from industrial, medical, and agricultural wastewater. The separation costs of feedstocks from wastewater before the electrolysis process are not considered in this techno‐economic analysis. In this techno‐economic evaluation, the model assumes that a facility can produce 1 ton of hydrogen daily by using 9 tons of water, based on the material balance equation (2H_2_O → 2H_2 _+ O_2_). For industrial water electrolysis, a 30% potassium hydroxide solution will be used, requiring the addition of 3.9 tons of potassium hydroxide. The cost of potassium hydroxide is set at $600, while the expense for water is considered negligible, as wastewater is utilized.^[^ ^81^ ^]^

(40) $$\begin{array}{c} \mathrm{Material}\;\mathrm{cost}\;\mathrm{of}\;\mathrm{electrolyte}\;\;\left({\$\;\mathrm{kg}}_{H2}^{-1}\right)=\\ 600\;\$\;\mathrm{to}n^{-1}*\;\frac{3.9\;\mathrm{ton}}{1000\mathrm{kg}}=2.34{\$\;\mathrm{kg}}_{H2}^{-1} \end{array}$$

### 3.2 Power Cost

Power cost is calculated by multiplying energy use (*W*) by the price of electricity. If we take the electricity price as $0.10 per kWh, this shows the highest cost for renewable energy at the moment.^[^ ^82^ ^]^ The energy consumption is derived using Equation (41):

(41) W=QU

The total charge, labeled as *Q*, needed by the electrolysis system to produce 1 ton of hydrogen is calculated. Here, *U* stands for the cell potential, which is taken to be 2 V. The charge is calculated using Equation (42).^[^ ^83^ ^]^

(42) $$Q=\frac{n\left(H_{2}\right)\times N\times F}{\mathrm{FE}}$$

where *n* stands for the amount of hydrogen, N represents the number of electrons involved in the water reaction (where *N* is 2), F indicates the Faraday constant (F equals 96 485 C mol⁻¹), and FE shows the Faraday efficiency of the system, which is considered to be 100%. Therefore, the total charge for the oxygen evolution reaction (OER) is:

(43) $$\left(Q\right)=\;\left(5*105\;*\;2\;*\;96485\right)/100\;\%=\;9.65*10^{10}$$

Total energy consumption for OER

(44) $$W=53.6{\;\mathrm{KWh}\;\mathrm{kg}}_{H2}^{-1}\;\;\;\;\;\;\;\;\;\;\;\;\;\;\;\;\;\;\;\;\;\;$$

The electricity cost for producing each kilogram of hydrogen is 5.36 $ $kg_{H2}^{-1}$, when the rate is 0.1 $ per kW. The reduced energy consumption relative (RRE) to the benchmark OER can be determined using Equation (45).

(45) $$\mathrm{RRE}\;=53.6\left(1-\frac{\left(\mathrm{Cell}\;\mathrm{voltage}\;\mathrm{of}\;\mathrm{the}\;\mathrm{OER}\;-\;\mathrm{Cell}\;\mathrm{voltage}\;\mathrm{of}\;\mathrm{waste}\;\mathrm{water}\;\mathrm{oxidation}\right)}{\left(\mathrm{Cell}\;\mathrm{voltage}\;\mathrm{of}\;\mathrm{the}\;\mathrm{OER}\right)}\right)\;$$

### 3.3 Capital Cost

The capital expenditure is divided into two main elements: the cost of the electrolysis cell and the expenses related to the membrane and catalyst. The dimensions and efficiency of the cell are primarily influenced by its operating current density, particularly in relation to the specified power consumption for production. To enhance the precision of capital cost estimation, particular assumptions are incorporated into the analysis, facilitating a more accurate and realistic calculation that corresponds with actual industry data. 1) The estimated cost for each square meter of the electrolysis cell is $10 000. The total expense for the catalyst and membrane represents 5% of the overall cost of the cell. 2) Considering the distribution of labor and facilities within the plant, a capacity factor of 0.8 is utilized, which suggests that the plant functions for 19.2 h each day. 3) The operating current density for the electrolysis cell is set at 0.3 A cm^−2^ at a voltage of 1.5 V, a standard value recognized in existing literature. The capital cost is calculated using Equations ((46), (47), (48)).

(46) $$\mathrm{Cell}\;\mathrm{cost}\;=\frac{10000Q}{T\;\;\times \;\;J}$$

(47) Membraneandcatalystcost=cellcost×5%

(48) $$\mathrm{Capital}\;\mathrm{cost}\;=\frac{\mathrm{Cell}\;\mathrm{cost}\;+\;\mathrm{Membrane}\;\mathrm{and}\;\mathrm{catalyst}\;\mathrm{cost}}{\mathrm{plant}\;\mathrm{life}\;}\;$$

where *J* is the operating current density of the electrolysis cell (*J* = 0.01 A cm^−2^), *T* is the daily running time of the factory (*T* = 19.2 h), the plant life assumed 30 years.

### 3.4 Maintenance Cost

The electrolysis cell requires daily maintenance, with the assumption that the associated maintenance expenses amount to 10% of the initial capital investment.

(49) Maintenancecost=10%×Capitalcost

### 3.5 Balance of Plant (BoP)

The Balance of Plant (BoP) primarily consists of expenses associated with a temperature control system, a circulating pump, a heating and insulation apparatus, and a storage tank. Importantly, these elements account for a substantial share of the initial investment, making up ≈30% of the overall capital expenditure.

(50) BOP=30%×Capitalcost

### 3.6 Installation Costs

This model exclusively considers the transportation and assembly of the electrolysis cell. Consequently, the installation cost for the factory is estimated utilizing the Lang Factor, which is ≈5.

(51) Installationcost=Capitalcost/LangFactor

### 3.7 Separation Cost

Hydrazine and urea do not yield any liquid value‐added products aside from hydrogen. Consequently, there are no separation costs associated with the electrolysis of wastewater for the separation of urea and hydrazine. The separation costs of value‐added products from the remaining wastewater containing amine, iodine, and alcohol through oxidation were taken into account. In a single electrolytic cell system, the expenses associated with separation and purification are significantly influenced by the activity and selectivity of both the anode and cathode reactions. Furthermore, the overall quality of the electrolytic cell, which encompasses the equipment, membrane, and catalyst, plays a critical role in determining these costs. It is important to note that the processes involved in product separation and purification are inherently complex in practical applications, with costs varying according to the specific type of reaction employed. To mitigate these separation expenses, one effective strategy is to enhance product concentration through high‐concentration electrolysis. Additionally, improving selectivity to reduce side reactions is essential. In this study, a comprehensive review of existing literature has revealed that the costs associated with product separation and purification play a significant role in the overall electricity expenditure for chemical processes. Specifically, the analysis indicates that in the case of iodine production, these costs are estimated to represent ≈20% of the total electricity expenditure. On the other hand, for amine oxidation product separation, the separation costs a significantly higher share, accounting for 40% of the total electricity usage. This disparity in cost allocation highlights the importance of considering the specific separation and purification requirements of different chemical processes when evaluating energy expenditure and operational efficiency. Understanding the impact of these costs on overall electricity consumption can guide process optimization strategies aimed at reducing energy usage and enhancing sustainability.

(52) Separationandpurificationcost=20%×Capitalcost

### 3.8 Operating Costs

In the context of a standalone electrolysis cell system, the operational expenses are predominantly focused on the oversight and adjustment of the electrolysis cell. This encompasses both personnel costs and continuous maintenance needs. As a result, it is suggested that the operational costs align closely with the maintenance expenses, representing ≈10% of the Total Capital Cost.

(53) Operating=10×Capitalcost

### 3.9 Total Cost

The total cost is the sum of all the above costs.

(54) $$\begin{array}{ccc} \mathrm{total}\;\mathrm{cost}\; & & \;\;\;\;\left(\mathrm{Plant}\;\mathrm{gate}\;\mathrm{levelized}\;\mathrm{cost}\right)=\;\mathrm{Material}\;\mathrm{cost}\;\\ & & +\;\;\mathrm{Power}\;\mathrm{cost}\;+\;\mathrm{Capital}\;\mathrm{cost}\;+\;\mathrm{Maintenance}\;\mathrm{cost}\;\\ & & +\;\mathrm{Balance}\;\mathrm{of}\;\mathrm{plant}\;+\;\mathrm{Installation}\;\mathrm{cost}\;\\ & & +\;\mathrm{Separation}\;\mathrm{cost}\;+\;\mathrm{Operating}\;\mathrm{cost} \end{array}$$

## 4 Market Size of Wastewater Electrooxidation Products

**Figure 8:**
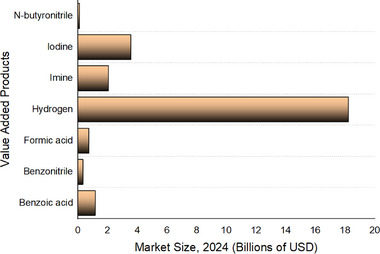
Market size of wastewater electrooxidation value‐added products.

The global benzoic acid market, valued at USD 1.17 billion in 2024, is projected to reach USD 1.77 billion by 2032, growing at a 5.30% CAGR (2025–2032). The benzonitrile market, worth USD 342.12 million in 2024, is expected to hit USD 360.72 million by 2032, with a 3.6% CAGR (2024–2032), driven by organic compound demand. The formic acid market reached 750 thousand tonnes in 2022 and is forecast to grow at a 4.48% CAGR until 2032.^[^ ^84^ ^]^ The hydrogen market, estimated at USD 18.23 billion in 2024, is projected to reach USD 47.83 billion by 2032, at a 10.4% CAGR. The imine market, valued at USD 2.07 billion in 2024, is expected to grow to USD 3.52 billion by 2032, at a 5.48% CAGR.^[^ ^85^ ^]^ The iodine market, worth USD 3.58 billion in 2023, is set to grow at a 4.4% CAGR (2024–2030), fueled by pharmaceutical and animal feed demand. The *N*‐butyronitrile market, valued at USD 0.12 billion in 2024, is projected to reach USD 0.20 billion by 2030, growing at a 6.8% CAGR (2024–2030) (**Figure** **8**).^[^ ^86^ ^]^

## 5 Typical Techno‐Economic Case Studies of Wastewater Electrolysis

The technoeconomic comparative analysis of anodic oxidation reactions (AOR), amino oxidation reactions (AmOR), hydrazine oxidation reactions (HzOR), iodide oxidation reactions (IOR), and urea oxidation reactions (UOR) aims to evaluate the feasibility of large‐scale hydrogen production through anodic oxidation processes utilizing alcohol, amine, hydrazine, iodide, or urea wastewater. The study reviews hydrogen generation rates of various catalysts across different oxidation mediators, covering electrolytes, maintenance, electricity, capital investment, separation processes, operational activities, installation, and BoP costs.

By examining relevant literature, the analysis provides insights into the catalytic efficiency and cost implications associated with each oxidation reaction type. The research delves into the intricacies of catalyst selection, operational parameters, and the impact of oxidation mediators on overall process economics. Through detailed cost modeling and formula application, the study offers a comprehensive assessment of the economic viability of hydrogen production via anodic oxidation of diverse organic substrates.

**Table** **2** serves as a reference point for the techno‐economic analysis, presenting a structured framework for evaluating the performance and cost‐effectiveness of catalysts in the context of AOR, AmOR, HzOR, IOR, and UOR. This comparative framework enables stakeholders to make informed decisions regarding the implementation of anodic oxidation processes for sustainable commercial‐scale hydrogen production. The study's findings may have significant implications for the development of efficient and cost‐competitive hydrogen generation technologies, highlighting the importance of selecting appropriate catalysts and oxidation mediators to optimize the overall economic feasibility of the process.

**Table 2:** Application and price of wastewater electro‐oxidation products.

No	Product	Importance	Price [$ kg^−1^]
1	Benzoic acid (C_7_H_6_O_2_)	Food, cosmetics, chemical industries	2.5
2	Benzonitrile (C_7_H_5_N)	Pharmaceutical, textiles, cosmetics	125
3	Formate (CH_2_O_2_)	Pesticide, Textile, and Tanning	1
4	Hydrogen (H_2_)	Energy	4
5	Imine (R_2_C═NR')	Dyes, polymers	50
6	Iodine (I_2_)	Medical	60
7	*n*‐butyronitriles (C_4_H_7_N)	Solar cells	75
8	Octanenitrile (C_8_H_15_N	Pharmaceutical, pesticides, and Perfume	50

A techno‐economic analysis of the complete system is conducted, incorporating various anodes (Au/CoOOH, MeCN, PW‐Co3N NWA/NF, CFP, and MOF‐Ni@MOF) for the coupling reactions of alcohols, amines, hydrazine, iodide, and urea oxidation are selected as representative cases, respectively. The chosen catalysts for techno‐economic analysis have demonstrated the highest catalytic efficiency in hydrogen production and the generation of value‐added products (**Table** **3**).

**Table 3:** Developed electrocatalysts for AOR, AmOR, HzOR, IOR, and UOR.

Electrocatalysts	Anolyte		Performance	Stability	Value‐added product selectivity	Refs.
Au/CoOOH	1 m KOH + 0.1 m benzyl alcohol	1.3 V@340 mA cm^−2^		10.5 h	Hydrogen (H_2_), Benzoic acid	[21]
Pt1/Ti0_0.8_W_0.2_N_x_O_y_	0.5 m methanol (MeOH) + 0.5 KOH	0.7 V@10 mA cm^−2^		NA	Hydrogen (H_2_), Formate (65%)	[46]
Co(OH)_2_@HOS/CP	3 m MeOH + 1 m KOH	1.385 V@10 mA cm^−2^		NA	Hydrogen (H_2_), Formate (100%)	[47]
NiO_x_	0.4 m BAS + butylamines	1.77 V@45 mA cm^−2^		50 h	Hydrogen(H_2_), *n*‐butyronitriles (95%)	[31]
Ni_2_P	1 m KOH + benzylamine	NA		–	Hydrogen (H_2_), Benzonitrile	[48]
MeCN	0.1 m NaClO_4 _+ MeCN	0.6 V@10 mA cm^−2^		–	Hydrogen (H_2_), Imine (90.7%)	[49]
PW‐Co_3_N NWA/NF	1 m KOH + 0.05 m Hydrazine	−55 mV@10 mA cm^−2^		25 h	Hydrogen (H_2_)	[37]
CC@CoNC‐600	1 M KOH + 0.5 Hydrazine	−61 mV@10 mA cm^−2^		50 h	Hydrogen (H_2_)	[50]
Ni_2_P/NF	1 m KOH + 0.5 Hydrazine	1 V@500 mA cm^−2^		10 h	Hydrogen (H_2_)	[51]
NiS_2_/TiM	1 m KOH + 0.5 Hydrazine	0.43 V @30 mA cm^−2^		10 h	Hydrogen(H_2_)	[52]
Ni(Cu)/NF	1 m KOH + 0.5 Hydrazine	0.41 V @100 mA cm^−2^		10 h	Hydrogen (H_2_)	[53]
Ni(Cu)@NiFeP/NM	1 m KOH + 0.5 Hydrazine	0.147 V@10 mA cm^−2^		12 h	Hydrogen (H_2_)	[54]
Cu_1_Ni_2_‐N/CFC	1 m KOH + 0.5 Hydrazine	0.24 V@10 mA cm^−2^		75 h	Hydrogen (H_2_)	[55]
CoP/TiM	1 m KOH + 0.5 Hydrazine	0.2 V@10 mA cm^−2^		–	Hydrogen (H_2_)	[56]
CoP/NCNT‐CP	1 m KOH + 0.5 Hydrazine	0.89 V @10 mA cm^−2^		10 h	Hydrogen (H_2_)	[57]
CoS_2_/TiM	1 m KOH + 0.5 Hydrazine	0.81 V @100 mA cm^−2^		–	Hydrogen (H_2_)	[58]
CoS_2_(Fe‐ CoS_2_)	1 m KOH + 0.1 Hydrazine	0.95 V@500 mA cm^−2^		40 h	Hydrogen (H_2_)	[59]
CoSe/CFP	1 m KOH + 0.5 Hydrazine	0.204 V@10 mA cm^−2^		–	Hydrogen (H_2_)	[60]
Cu_3_P/CF	1 m KOH + 0.5 Hydrazine	0.72 V@100 mA cm^−2^		–	Hydrogen (H_2_)	[61]
FeP NA/NF	1 m KOH + 0.5 Hydrazine	0.5 V @125 mA cm^−2^		–	Hydrogen (H_2_)	[62]
D‐MoP/rGO	1 m KOH + 0.5 Hydrazine	0.74 V @100 mA cm^−2^		12 h	Hydrogen (H_2_)	[63]
CoP/NCNT‐CP	1 m KOH + 0.5 Hydrazine	0.65 V@80 mA cm^−2^		10 h	Hydrogen (H_2_)	[64]
CFP	Anode:0.1 m HClO_4_ + 1 m NaI	0.53 V@10 mA cm^−2^		18 h	Hydrogen (H_2_), Iodine (93%)	[41]
MoS_2/_C	–	0.63 V @10 mA cm^−2^		200 h	Hydrogen (H_2_), Iodine (NA)	[41]
Pt/C (20%)	Anode:0.1 m HClO_4_ + 1 m NaI	0.75 V @10 mA cm^−2^		–	Hydrogen (H_2_), Iodine (100%)	[65]
Coke with graphite	Anode: 2.5 mm KI + 0.25 m KCl + Coke	0.76 V @10 mA cm^−2^		–	Hydrogen (H_2_), Iodine (65%)	[66]
Mo‐N_4_/d‐C	Anode:0.1 m HClO_4_ + 1 m NaI	0.77 V@10 mA cm^−2^		100 h	Hydrogen (H_2_), Iodine (100%)	[65]
RuTiO‐550	Anode:0.1 m HClO_4_ + 1 m NaI	1.01 V@10 mA cm^−2^		10 h	Hydrogen (H_2_), Iodine (NA)	[67]
RuSn SAO	Anode:0.1 m HClO_4_ + 1 m NaI	1.07 V @10 mA cm^−2^		30 h	Hydrogen (H_2_), Iodine (NA)	[68]
NKX‐2697dyes on TiO_2_	Anode: 0.1 m KI + acetonitrile−ethanol	1.19 V @10 mA cm^−2^		–	Hydrogen (H_2_), Iodine (NA)	[69]
MOF‐Ni@MOF‐Fe	1 m KOH + 0.5 m urea	1.346 V @10 mA cm^−2^		10 h	Hydrogen (H_2_)	[70]
Ni‐MOF	1 m KOH + 0.33 m urea	1.36 V @10 mA cm^−2^		10 h	Hydrogen (H_2_)	[71]
Zn_0.8_Co_0.92_P/TM	1 m KOH + 0.5 m urea	1.38 V@10 mA cm^−2^		10 h	Hydrogen (H_2_)	[72]
Ni‐Mo nanotube	1 m KOH + 0.1 m urea	1.43 V @10 mA cm^−2^		10 h	Hydrogen (H_2_)	[73]
Fe‐Ni_3_S_2_/Ni foam	1 m KOH + 0.33 m urea	1.46 V @10 mA cm^−2^		20 h	Hydrogen (H_2_)	[74]
Ni/C	1 m KOH + 0.33 m urea	1.6 V @10 mA cm^−2^		12 h	Hydrogen (H_2_)	[75]
MnO_2_/MnCo_2_O_4_/Ni	1 m KOH + 0.5 m urea	1.75 V @10 mA cm^−2^		15 h	Hydrogen (H_2_)	[76]

Abbreviations: Au/CoOOH, Gold nanoparticles (NPs) on cobalt oxyhydroxide nanosheets; Pt1/Ti00.8W0.2NxOy, Pt single atom dispersed on dual doped TiO2 (Pt1/ Ti0.8W0.2NxOy); Co(OH)2@HOS/CP, Cobalt Hydroxide@Hydroxysulfide Nanosheets; NiOx, Nickel Oxide; Ni2P, nickel phosphide nano meshes; MeCN, Methyl Cyanide; Ni/C, Pomegranate‐like Nickel on Carbon; PW‐Co3N NWA/NF, Phosphorus and Tungsten co‐doped co nanowire arrays; CC@CoNC, Cobalt/Nitrogen‐codoped Carbon nanosheet arrays on carbon cloth; Ni2P/NF, Nickel phosphide nanoarrays grown in situ on nickel foam; NiS2/TiM, Nickel sulfideNiS2 nanosheet array on Titanium mesh; CoP/NCNT‐CP, Cobalt phosphide nanoparticles embedded into Nitrogen‐doped Carbon nanotubes, grafted on Carbon Polyhedron; Ni(Cu)/NF, Nickel Copper nano‐fibers; Ni(Cu)@NiFeP/NM, Ni(Cu)@NiFeP onto nickel foam (NM); Cu1Ni2‐N/CFC, Copper–nickel nitride on Carbon fiber cloth; CoP/TiM, Cobalt phosphide on Titanium mesh; CoS2/TiM, Cobalt Sulfide on Titanium mesh; CoP/NCNT‐CP, Cobalt phosphide nanoparticles embedded into N‐doped carbon nanotubes; CoS2(Fe‐ CoS2), Iron doped Cobalt Sulfide; CoSe/CFP, Cobalt Selenide nanosheet arrays supported on Carbon fiber paper; Cu3P/CF, Cupric Phosphide on Carbon fiber; FeP NA/NF, Iron Phosphide Nanosheets Array; D‐MoP/rGO, Defective crystalline molybdenum phosphides; CoP/NCNT‐CP, Cobalt Sulfide on N‐doped carbon nanotubes; CFP, Carbon fiber paper; MoS2/C, Molybdenum Sulfide on Carbon; Pt/C, Platinum on Carbon; Mo‐N4/d‐C, Nitrogen coordinated Molybdenum on defective carbon Nitride; RuTiO, Ruthenium‐Titanium alloy Oxide; RuSn SAO, Ruthenium–Tin surface alloy Oxide; NKX‐2697dyes on TiO2, NKX‐2677 dye on Titanium Oxide; MOF‐Ni@MOF‐Fe, Ni/Fe bimetallic organic framework;  Ni‐MOF, Nickel Metal Organic frame work; Zn0.8Co0.92P/TM, Zinc on Cobalt Phosphide; Ni‐Mo, Nickel Molybdenum nano‐tube; Fe‐Ni3S2/Ni foam, iron–nickel sulfide nanoarrays on Nickel foam; Ni/C, Nickel on Carbon; MnO2/MnCo2O4/Ni, Manganese Oxide layered on Manganese cobalt Oxide, supported by a Nickel foam.

**Figure 9:**
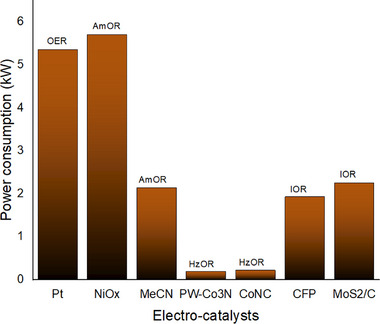
Power consumptions of different wastewater oxidation electro catalysts.

**Figure 10:**
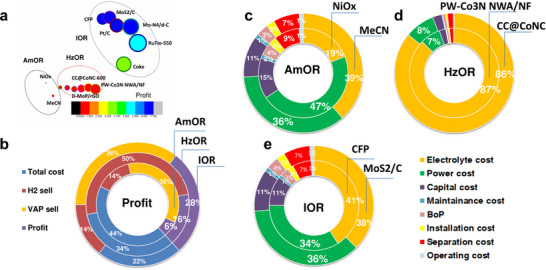
Techno‐economic analysis (TEA) a) Profit analysis of various catalysts. b) Revenue of amine oxidation reaction (AmOR), Hydrazine oxidation reaction (HzOR), and iodide oxidation reaction (IOR). c) Amine oxidation reaction (AmOR) catalysts H_2_ and value‐added product (VAP) production cost. d) Hydrazine oxidation reactions (HzOR) catalysts H_2_ production cost. e) Iodide oxidation reaction (IOR) catalysts H_2_ and value‐added product (VAP) production cost.

**Figure 11:**
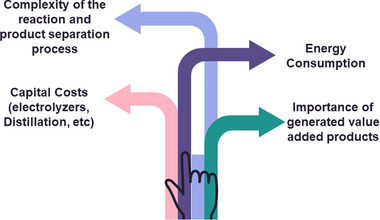
Important criteria for selections of feedstock chemicals for value‐added water electrolysis process.

The oxidation process of amine wastewater results in the production of benzonitrile, a notably expensive by‐product, facilitated by the use of nickel oxide (NiOx) as an electrocatalyst. This reaction requires an energy input of 5.72 kW (as illustrated in **Figure** **9**) and is characterized by complex side reactions, which contribute to a low yield of the desired product. The cost associated with separation will account for 9% of the overall expenditure (**Figure** **10**c). The energy consumption associated with hydrazine wastewater is minimal, ranging from 0.2 to 0.21 kW, as illustrated in Figure 9. This represents ≈7–8% of the overall expenditure, as shown in Figure 10d. However, the only byproduct generated is hydrogen, which considerably diminishes the potential for profitability. The oxidation reaction of iodide wastewater, represented by the equation (I^−^  +  4H_2_O → I_2_  +  4H_2_  +  I_3_^−^  +  IO_4_^−^), yields 1 kg of hydrogen (H_2_) and 0.25 kg of iodine (I_2_). The power consumption for the iodide oxidation reaction (IOR) utilizing a CFP electrode is measured at 1.93 kW, while the MoS_2_/C electrode exhibits a higher power consumption of 2.25 kW. As illustrated in Figure 10e, the cost distribution for various components when employing the CFP electrocatalyst is as follows: electrolyte cost at 41%, power cost at 34%, capital cost at 11%, maintenance cost at 1%, installation cost at 3%, separation cost at 2%, and operating cost at 7%, with an additional 1% for other expenses. The separation cost is notably the highest due to the challenges associated with isolating I_2_ and *I*_3_^−^, which necessitates increased power consumption and associated costs (**Figure** **11**).

Upon reviewing all relevant factors, the total cost of producing hydrogen and value‐added products (VAP) for AOR, AmOR, HzOR, IOR, and UOR is calculated to be $6.37 per kg of H_2_, $6.06 per kg of H_2_, $2.68 per kg of H_2_, $5.69 per kg of H_2_, and $10.69 per kg of H_2_, respectively (see Figure S1, Supporting Information). Nevertheless, the hydrogen production costs associated with AOR and UOR are deemed unviable. Iodine, hydrazine, and amine wastewater oxidation generate 28%, 16%, and 6% profit compared to total cost respectively. Although amine wastewater oxidation gives high valuable products, the problem for revenue generation is highly dependent on the cost of product separation and product quality.

**Figure 12:**
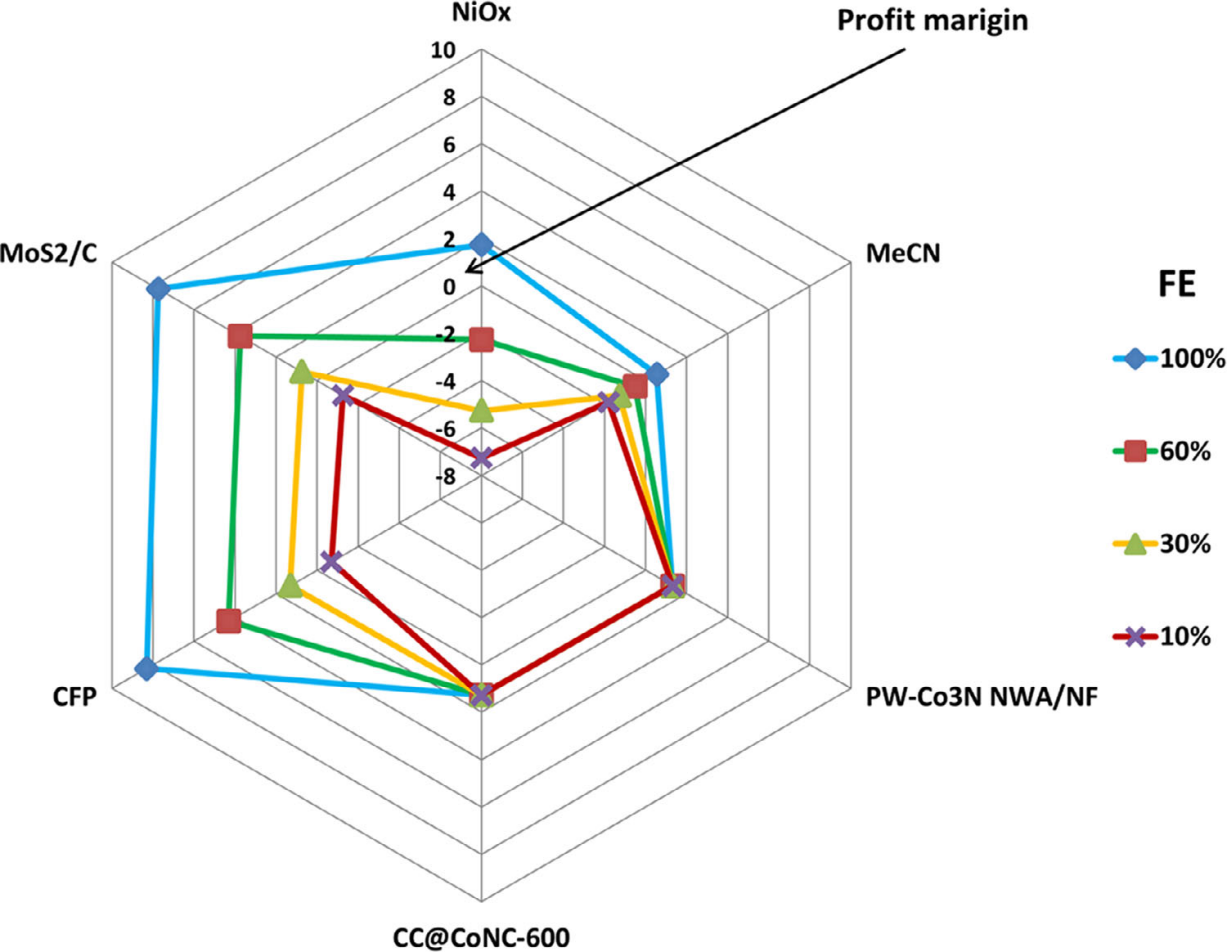
Profit sensitivity analysis of different catalysts with varying faradaic efficiency (FE).

During the practical electrolysis process of the coupling system, competition for OER at the coupling anode inevitably results in diminished electrolysis efficiency. Additionally, energy losses occurring during large‐scale electrolysis processes or series operations further amplify the system's overall efficiency reduction. Consequently, the electrolysis system's efficiency must be incorporated into sensitivity analysis (**Figure** **12**). The sensitivity analysis is used for hydrogen production through wastewater electrolysis. The economic viability of the system is examined with different Faraday efficiencies (FE) for VAP, specifically at 10%, 30%, 60%, and 100%. We assume that the FE of H_2_ is 100% for all setups. Sensitivity analysis indicates that iodine catalysts, specifically CFP and MoS_2_/C, demonstrate profitability at faradaic efficiencies (FE) of up to 30%. In contrast, amine catalysts, such as MeCN and NiOx, exhibit profitability at faradaic efficiencies greater than 60%. However, certain hydrazine catalysts, namely PW‐Co3N NWA/NF and CC@CoNC‐600, are not influenced by faradaic efficiency, because they do not produce value‐added products.

## 6 Conclusion‐and‐Future Perspectives

This review article delves into the significant advancements in the field of electrooxidation for the sustainable production of hydrogen and value‐added chemicals through the coupling of small molecules (alcohol, amine, hydrazine, iodine, and urea) in wastewater treatment processes. By integrating green electrooxidation technologies, the energy efficiency of hydrogen production is enhanced while offering a green pathway for the production of valuable chemicals or the degradation of pollutants. This review not only highlights the developments in wastewater anodic oxidation electrocatalysts but also provides a comprehensive techno‐economic analysis (TEA) to showcase the economic viability of these sustainable approaches. The techno‐economic analysis indicates that, despite the sustained high prices of global power generation, the wastewater electro‐oxidation system is capable of producing hydrogen at a competitive cost. The electrochemical wastewater oxidation presents a remarkable energy transformative approach and opportunity for industries and simultaneously broadens the diversity of product offerings within these sectors. However, the following future perspectives should be into account for the affordable hydrogen generation from a wastewater electrolysis system.

### 6.1 Tecno‐Economic Analysis of Other Oxidation Reactions

Conducting a detailed techno‐economic analysis on emerging alternative electrooxidation reactions such as tetrazoles, furazans, quinolines, ascorbic acid, sterol, trimethylamine, etc. is crucial for evaluating their feasibility for industrial applications. This analysis involves assessing the economic viability and technical feasibility of these reactions to determine their potential for commercialization. Key concepts to consider include understanding the reaction mechanisms, optimizing process conditions, estimating production costs, evaluating market demand, and comparing the results with existing electrooxidation processes. Original insights can be gained by exploring the scalability, sustainability, and competitiveness of these alternative reactions compared to traditional methods. By conducting a thorough techno‐economic analysis, valuable insights can be gained to guide decision‐making in adopting these emerging electrooxidation reactions in industrial processes. The subsequent future directions for additional research are presented below.

### 6.2 Catalyst Design

Catalyst design strategies today mainly come from traditional water electrolysis, and there is still a gap in targeted catalyst design for wastewater small‐molecule oxidation. To address this, it is important to thoroughly understand the catalytic reaction mechanisms and create targeted designs for each small‐molecule oxidation reaction. Since small molecules often have various groups, understanding how these groups interact with catalysts is crucial for developing highly efficient catalysts. This will help maximize the catalyst's potential.

### 6.3 Value‐Added Products Separation and Purification

Product separation constitutes a critical operational phase in the practical aspects of chemical production. However, most prior studies on wastewater electrolysis predominantly emphasize the development of highly efficient electrocatalysts and the generation of value‐added products. Consequently, the challenges associated with the “post‐reaction” phase, including product separation and the economic feasibility of the overall system, are frequently neglected. Therefore, future research and industrial implementation should prioritize optimizing product separation techniques to enhance the effectiveness and economic feasibility of chemical production through electrolysis. By addressing this gap in current practices, the field can advance toward more sustainable and efficient processes, bringing us closer to achieving a greener future in hydrogen and chemical manufacturing.

For example, the challenge of separating iodine and triiodide iodide wastewater electrooxidation products stems from their similar chemical properties, especially their similar solubilities in water. This similarity makes traditional separation methods, such as precipitation or filtration, ineffective and costly. To overcome this issue, innovative separation techniques must be explored. One promising approach is ion exchange chromatography, which can exploit the differences in the charge and size of iodine and triiodide ions to selectively separate them. Additionally, advanced oxidation processes, such as ozonation or UV irradiation, can be used to degrade one of the compounds into simpler byproducts that are easier to separate. Integrating these novel techniques into iodide wastewater oxidation processes can help reduce the separation cost and improve the efficiency of iodine and triiodide iodide separation.

### 6.4 Self‐Powered Wastewater Anodic Oxidation

Reducing the expenses associated with hydrogen production could potentially be addressed through self‐powered anodic oxidation. This method entails the electrochemical transformation of wastewater into hydrogen (H_2_) and other beneficial byproducts, all without requiring an external power source. For instance, the decoupled hydrazine system has been suggested to facilitate adaptable energy conversion and storage. This system harnesses solar energy to promote high‐rate hydrogen production during the day, while at night, it utilizes hydrazine oxidation to generate electricity through the p‐VHCF‐N_2_H_4_ liquid battery.^[^ ^87^ ^]^

### 6.5 Resource Recovery

The prospects for electrolysis of real wastewater should emphasize practical applications in addressing environmental pollution by considering metal recovery. Real wastewater often contains valuable metals that can be recovered during the electrolysis process. This not only reduces the environmental impact of metal pollution but also provides economic benefits by reclaiming valuable resources.

## Conflict of Interest

The authors declare no conflict of interest.

## Supporting information



Supporting Information
