# Supplementary material for: Waste to Wealth: Electrochemical Innovations in Hydrogen Production From Industrial Wastewater
Source: Glob Chall. 2025 Apr 25;9(6):2500043. doi: 10.1002/gch2.202500043 (PMC12151803; doi:10.1002/gch2.202500043)
Supplement: Supplementary file 1 — Supporting Information [file GCH2-9-2500043-s001.docx]

**Waste to Wealth: Electrochemical Innovations in Hydrogen Production from Industrial Wastewater**

Tesfaye Alamirew Dessie^1^, Lemlem Seyoum Seifu^1^, Woldesenbet Bafe Dilebo^1,4^

^1^Faculty of Chemical and Food Engineering, Bahir Dar Institute of Technology, Bahir Dar University, Bahir Dar 79, Ethiopia.

^3^NanoElectrochemistry Laboratory, Department of Chemical Engineering, National Taiwan University of Science and Technology, Taipei 10607, Taiwan

^4^Sustainable Electrochemical Energy Development Center, National Taiwan University of Science and Technology, Taipei 10607, Taiwan.


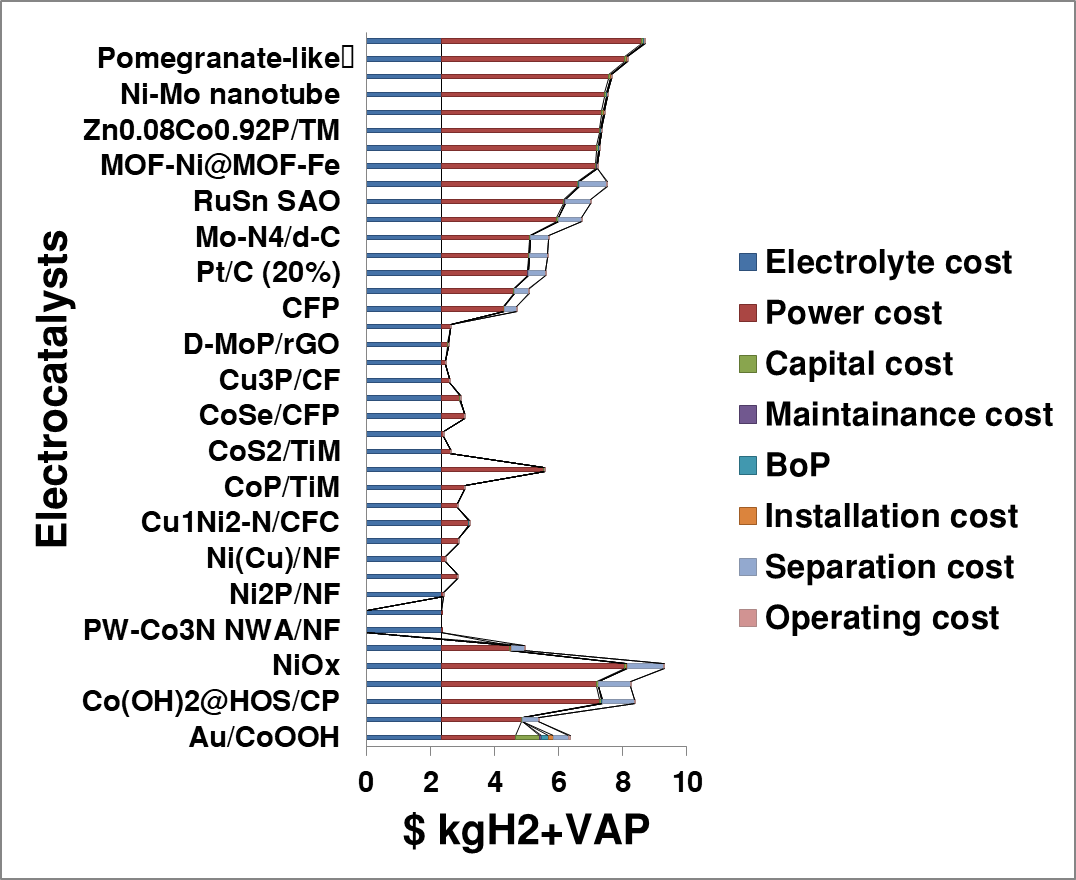


**Figure S1**: Plant gate levelized cost





**Figure S2**: Profitability analysis of various electro-catalysts
